# Targeting melanoma stem cells with the Vitamin E derivative δ-tocotrienol

**DOI:** 10.1038/s41598-017-19057-4

**Published:** 2018-01-12

**Authors:** Monica Marzagalli, Roberta Manuela Moretti, Elio Messi, Marina Montagnani Marelli, Fabrizio Fontana, Alessia Anastasia, Maria Rosa Bani, Giangiacomo Beretta, Patrizia Limonta

**Affiliations:** 1 Department of Pharmacological and Biomolecular Sciences, Università degli Studi di Milano, Milano, 20133 Italy; 2 Laboratory of Biology and Treatment of Metastasis, IRCCS-Mario Negri Institute for Pharmacological Research, Milano, 20156 Italy; 3 Department of Pharmaceutical Sciences, Università degli Studi di Milano, Milano, 20133 Italy

## Abstract

The prognosis of metastatic melanoma is very poor, due to the development of drug resistance. Cancer stem cells (CSCs) may play a crucial role in this mechanism, contributing to disease relapse. We first characterized CSCs in melanoma cell lines. We observed that A375 (but not BLM) cells are able to form melanospheres and show CSCs traits: expression of the pluripotency markers SOX2 and KLF4, higher invasiveness and tumor formation capability *in vivo* with respect to parental adherent cells. We also showed that a subpopulation of autofluorescent cells expressing the ABCG2 stem cell marker is present in the A375 spheroid culture. Based on these data, we investigated whether δ-TT might target melanoma CSCs. We demonstrated that melanoma cells escaping the antitumor activity of δ-TT are completely devoid of the ability to form melanospheres. In contrast, cells that escaped vemurafenib treatment show a higher ability to form melanospheres than control cells. δ-TT also induced disaggregation of A375 melanospheres and reduced the spheroidogenic ability of sphere-derived cells, reducing the expression of the ABCG2 marker. These data demonstrate that δ-TT exerts its antitumor activity by targeting the CSC subpopulation of A375 melanoma cells and might represent a novel chemopreventive/therapeutic strategy against melanoma.

## Introduction

Cutaneous melanoma is one of the most prevalent cancers in the caucasian population; its incidence has increased faster than other tumors during the last three decades, particularly in young females^1^.

The majority of melanomas are diagnosed in the early stage, when they are treatable with surgical resection and with IFN-α-2b with a high five-year survival rate^2^. However, the prognosis of late stage metastatic melanoma is still extremely poor. For metastatic melanoma, chemotherapeutic agents, dacarbazine or temozolomide, have been considered the reference drugs; however, patients very often become resistant to these compounds, with low overall response and survival rates^3^.

Approximately 50% of cutaneous melanomas harbor an activating mutation in the BRAF protein (valine at codon 600 is substituted by glutamic acid, V600E), leading to constitutive activation of the mitogen-activated protein kinase (MAPK) pathway involved in cell growth; other V600 mutations in BRAF were shown to correlate with melanoma development. NRAS mutations were reported in about 30% of patients and shown to be associated with increased activation of two main signaling pathways: the PI3K/Akt and the MAPK cascades^4^. Based on these observations, targeted drugs were introduced in melanoma therapy. Selective inhibitors of V600E BRAF mutated melanoma (vemurafenib, dabrafenib) were reported to improve the survival of patients harboring this specific mutation. However, a rapid development of tumor resistance was observed after these treatments and was found to be related to the BRAF-independent activation of MEK. Combining selective mutation-specific BRAF and MEK inhibitors (trametinib), was shown to improve the response rate and progression-free survival in patients with advanced melanoma^5^. Novel BRAF inhibitors with selective MEK inhibitor activity have also been suggested for the treatment of NRAS or BRAF mutant melanomas^6^.

Another modality in the treatment of aggressive melanoma involves the use of immunotherapy, such as IL-2^7^. More recently, immune checkpoint inhibitors have been used to treat melanoma. Antibodies against cytotoxic T lymphocyte antigen 4 (CTLA-4), such as ipilimumab, and programmed cell death receptor 1 (PD-1), such as nivolumab and pembrolizumab, were developed and stimulated renewed enthusiasm for anticancer immunotherapy^8^; however, these compounds did not show the expected improvement in overall survival since they are associated with a potential toxicity. The combination of CTLA-4 and PD-1 inhibitors has led to better results than the two monotherapies alone^9^.

Further studies aimed at defining the sequencing, duration and combinations of targeted and immune check point inhibitor therapies are at present ongoing^10^; these studies are necessary for the improvement of the outcome of late stage melanoma patients.

The development of resistance to previously effective treatments is at present a serious challenge for patients undergoing cancer therapy, including melanoma patients. Innate and acquired chemoresistance of most tumors after treatment with conventional chemotherapeutic/molecular targeted agents accounts for the majority of relapse cases in cancer patients. Chemoresistance is due to multiple key molecular players: activation of proliferative/survival signaling pathways such as the epidermal growth factor receptor (EGFR) family members and their associated intracellular pathways (ERK and PI3K pathways); dysfunction or loss of apoptosis pathways; increased expression/activity of multidrug resistance mechanisms; modification of drug targets and inhibition of tumour suppressor genes that triggers DNA methylation pathways; triggering of protective autophagy; altered expression of microRNAs (miRNAs) and other non-coding RNAs (ncRNAs).

On the other hand, it is now well accepted that also cancer stem cells (CSCs) are deeply involved in the development of therapy resistance, thereby contributing to disease relapse after an initial positive response to therapy^11,12^. An early definition implies that tumors are a mixture of malignant stem cells and their differentiated daughter cells: actually, the classical notion supports that cancer stem cells (CSCs) are characterized by their limited number and their ability for self-renewal through asymmetric cell division. According to the hierarchical model of tumor progression, CSC is a tumor cell that has the capacity for self-renewal, the ability to generate all heterogeneous tumor cell lineages, giving rise to the bulk of the tumor mass and to recapitulate continuous tumor growth^13^. CSCs are usually identified on their ability to generate tumorspheres in suspension cultures, to possess high invasive behavior, to give rise to the heterogeneous original tumor when inoculated in nude mice, and to express specific surface markers^14^. However, the reliability of these markers in different tumors, including melanoma, has recently become a serious matter of debate^15,16^. In epithelial cancer cells, Miranda-Lorenzo and coworkers identified cells with high autofluorescence levels showing CSCs features and phenotypes. In these cells, autofluorescence was reported to be due to the presence of riboflavin (vitamin B2) in membrane-bounded cytoplasmic vesicles coated with the ATP-dependent transporter ABCG2. The authors conclude that autofluorescence can be considered a reliable marker to be used to isolate CSCs from the bulk of a tumor mass^17^.

Given their role in drug resistance and tumor relapse, identifying and characterizing CSCs is crucial for the development of novel and more effective therapeutic approaches, aimed to target this aggressive subpopulation of cancer cells. So far, different therapeutic approaches specifically targeting the CSCs subpopulation have been developed for different types of tumors^18,19^, including melanoma^20^. Interestingly, natural compounds previously shown to possess anticancer activity were also reported to specifically target CSCs^21–25^.

Vitamin E is composed of eight compounds: α, β, γ and δ tocopherols, and the four corresponding tocotrienols (TTs)^26^. TTs (specifically, γ-TT and δ-TT) were widely reported to be associated with significant health benefits in different chronic diseases, based on their neuroprotective, cholesterol-lowering, antioxidant and antiinflammatory activites^27,28^. TTs were also shown to possess antitumor activity by suppressing cancer cell proliferation and by increasing the sensitivity of tumor cells to chemotherapeutic agents^29,30^. We recently reported that δ-TT exerts a significant proapoptotic activity in human cutaneous melanoma cells (while sparing normal melanocytes), independently on their genetic mutations, by triggering the endoplasmic reticulum (ER) stress prodeath pathways^31^.

Aim of this study was to identify and characterize the subpopulation of CSCs in melanoma cells and to investigate whether δ-TT might affect melanoma cell growth by targeting this aggressive subpopulation of cancer cells.

## Results

### Identification of stem cell-like subpopulations in human melanoma cell lines

**Figure 1:**
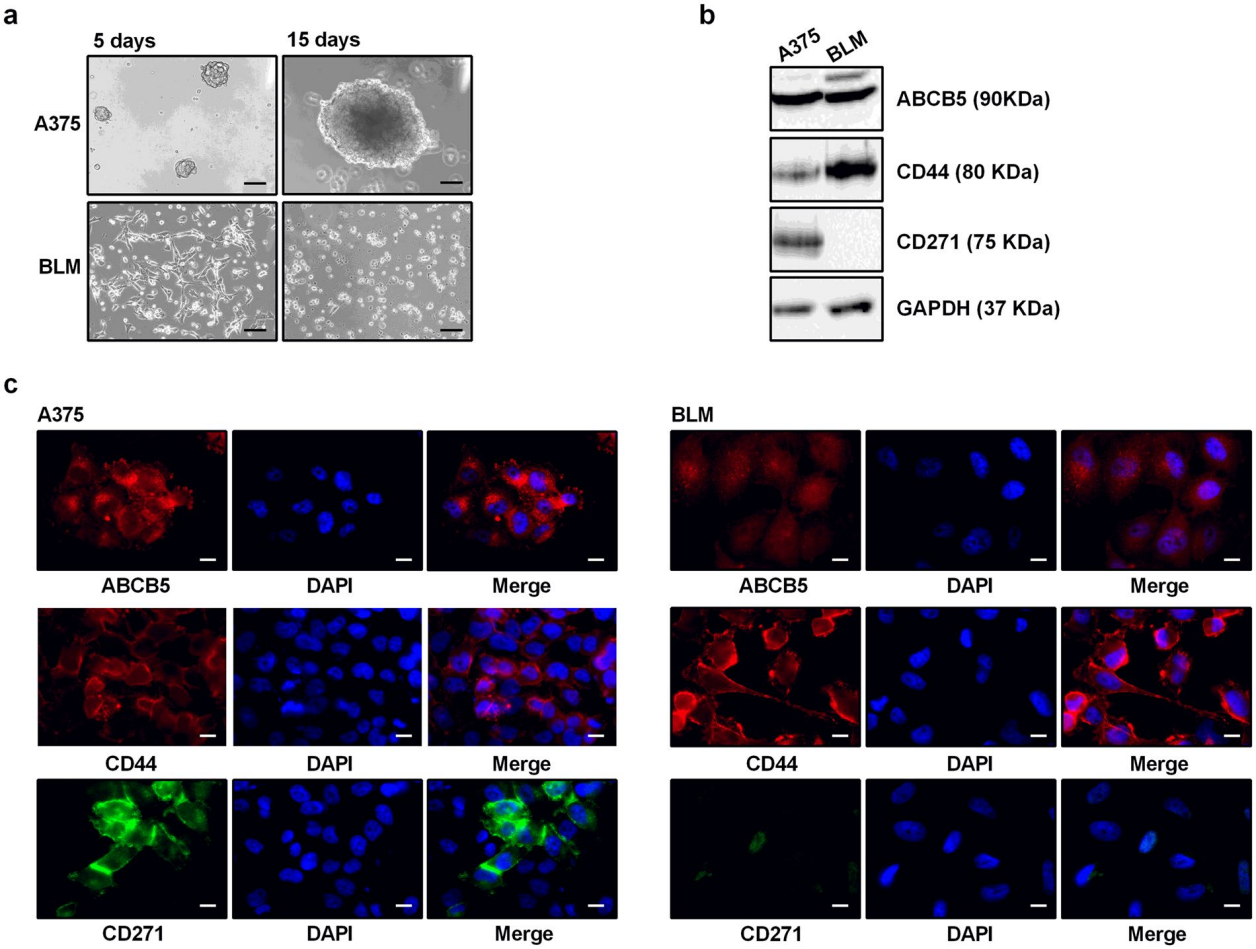
Identification of stem cell-like subpopulations in human melanoma cell lines. (**a**) The spheroidogenic potential of A375 and BLM cells was assessed by plating cells in stem cell medium (Euromed-N), supplemented with 10 ng/ml EGF, 10 ng/ml FGF, 1% N2. Melanosphere growth was monitored for up to 15 days, and cells were photographed at different time points with a 10 × /1.4 objective lens (scale bar, 30 μm). (**b**) The protein expression of three melanoma stem cell markers was assessed by Western blot analysis. GAPDH expression was evaluated as a loading control. One representative of three different experiments, for each analysis performed, is shown. Cropped gels/blots are displayed. Original uncropped Western blots were reported in the Supplementary Figure S1A. (**c**) Immunofluorescence analysis was performed in order to verify the pattern of distribution of ABCB5, CD44 and CD271 in A375 and BLM cells. Cells were photographed with a 63 × /1.4 objective lens (scale bar, 5 μm). One representative of three different experiments is shown.

A typical feature of cancer stem cells is their ability to form 3D tumorspheres when cultured in the appropriate stem cell medium^32^. To assess the presence of a CSC subpopulation in human melanoma cell lines, A375 and BLM cells were plated as single cell-suspension and cultured in serum-free Euromed-N medium supplemented with growth factors (10 ng/ml EGF and 10 ng/ml FGF-2) and 1% N2; their ability to form melanospheres was followed and images were taken after 5 and 15 days of culture. We found that A375 cells, but not BLM cells, were able to form melanospheres at both time intervals. In particular, at 15 days of culture, these spheres appear as compact cell aggregates, characterized by a well-rounded shape and regular edges (Fig. 1a).

Another feature of CSCs is the specific and differential expression of surface markers with respect to their non-stem counterpart^33^. In A375 and BLM cells, we analyzed the expression of three surface markers, previously shown in the literature to be related to a subpopulation of melanoma cells endowed with self-renewal ability: ABCB5, a member of the ATP-binding cassette (ABC) transporter family known to be involved in drug efflux, detected in tissues derived from the neuroectodermal lineage, including melanocyte progenitors; CD44, an ubiquitous cell surface glycoprotein involved in cell migration and considered one of the most consistent CSC markers; CD271, also known as a low-affinity nerve growth factor receptor (NGFR) or p75NTR, a marker of neural crest cells representing a de-differentiation marker for melanoma cells^34,35^. By Western blot analysis we could observe that ABCB5 and CD44 are expressed in both melanoma cell lines; conversely, CD271 is expressed in A375 cells, while no protein band could be detected in BLM cells (Fig. 1b). Immunofluorescence analysis confirmed these observations, showing that ABCB5 and CD44 are ubiquitously expressed in both A375 and BLM cells, while CD271 is expressed only in A375 and, specifically, it shows a differential pattern of expression in different cells (Fig. 1c).

We concluded that a subpopulation of cancers cells able to form tumorspheres is present in the A375 (but not in the BLM) cell line and we hypothesized that CD271 might be considered as a putative melanoma stem cell marker for this cell line. The inability of BLM cells to form spheres makes it difficult to study the anticancer effects of drugs on the CSC subpopulation in this cell line. Thus, based on these observations, further experiments were performed in the A375 melanoma cell line. On the other hand, it cannot be excluded that a subpopulation of CSCs with different markers of stemness may be present in the BLM cell line. Experiments are at present undergoing in the authors’ laboratory to investigate this issue.

### A375 spheroid-derived cells show CSCs traits

**Figure 2:**
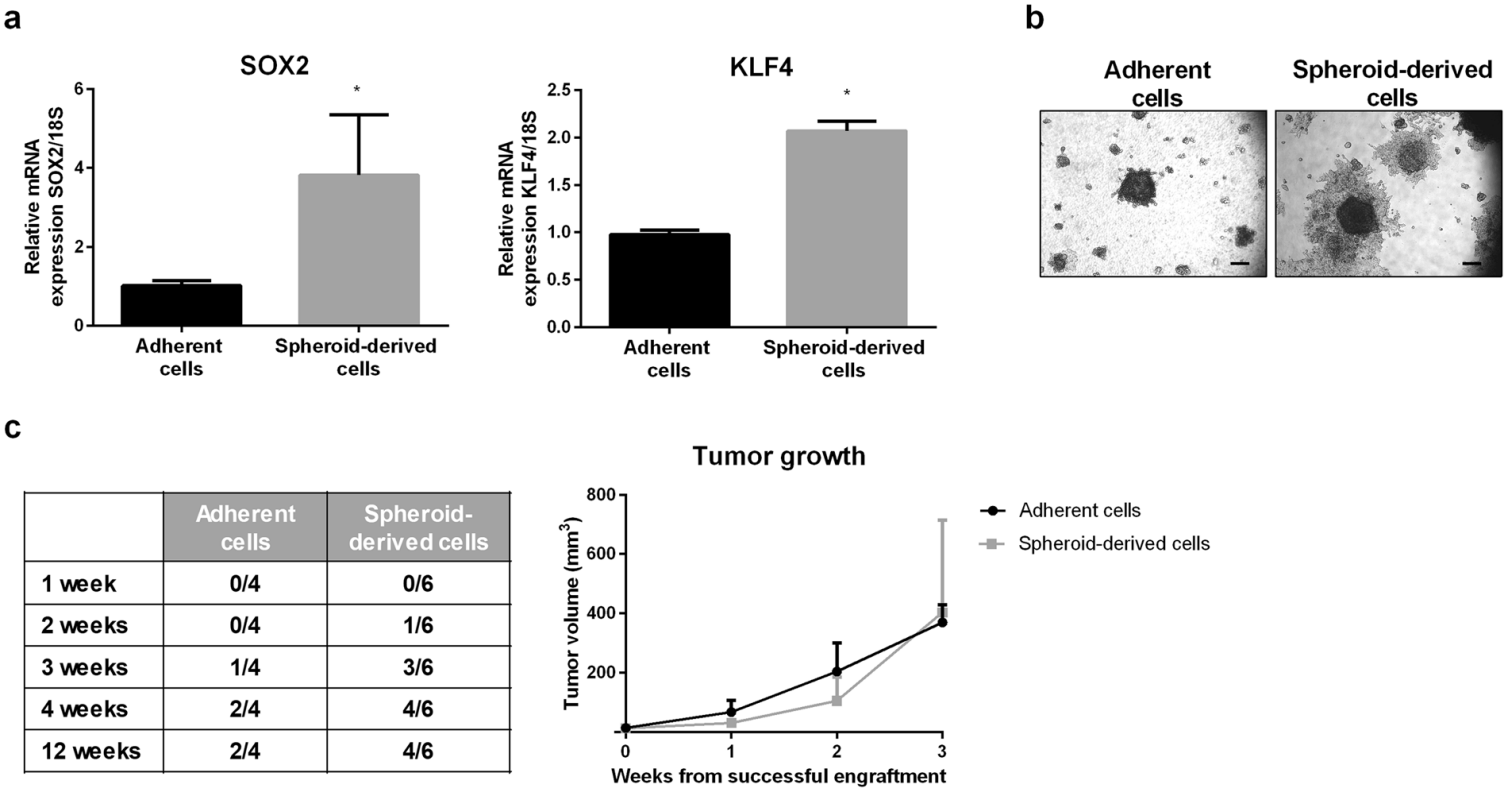
A375 spheroid-derived cells show CSCs traits. (**a**) The mRNA expression levels of the two embryonic stem cell markers SOX2 and KLF4 were measured by real-time RT-PCR, in both adherent and spheroid-derived cells. Three replicates were performed, and the experiment was repeated three times. Data were analyzed by unpaired t-test, and represent mean values ± SEM (*p < 0.05 vs Adherent cells). (**b**) The invasive ability of adherent and spheroid-derived cells was assessed by Matrigel invasion assay. Cells were seeded on Matrigel-coated plates and left to grow for up to 15 days. Cells were then photographed with a 4 × /1.4 objective lens (scale bar, 75 μm). One representative of three different experiments is shown. (**c**) The *in vivo* capability to originate tumors was assessed by transplantation of adherent or spheroid-derived cells (5 × 10^5^) into immunodeficient mice. Tumor take was assessed by palpation (twice/week) for up to 12 weeks (left panel). The growth rate of engrafted and generated tumors (n = 2 in adherent and n = 4 in spheroid-derived cells) was monitored weekly by measuring tumor size and calculating tumor volume (mm^3^) as [length (mm) x width^2^ (mm^2^)/2] (right panel).

To demonstrate the enrichment in CSCs in the spheroid cultures, we analyzed the expression of pluripotent embryonic stem cell markers in A375 spheroids and the invasive ability of these cells. By RT-real time PCR analysis, we could observe that the expression of the embryonic transcription factors SOX2 and KLF4 is significantly increased in spheroid-derived cells, when compared to A375 adherent melanoma cells (Fig. 2a).

Since CSCs have been shown to be more invasive than their adherent counterpart, we analyzed the invasive properties of A375 spheroid cultures. As shown in Fig. 2b, Matrigel invasion assays demonstrated that, after 15 days of culture on Matrigel-coated plates, spheroid-derived cells have a higher invasive capacity than the adherent counterpart.

To further confirm that melanospheres represent a subpopulation of A375 cells endowed with stem cell-like properties, we evaluated the tumor initiation potential of these spheres *in vivo*. Melanosphere-derived and adherent cells were subcutaneously injected in immunodeficient mice. Tumor take was assessed by palpation (twice a week up to 12 weeks) and growth of successfully engrafted tumors was monitored weekly by measuring tumor size.

We found that spheroid-derived cells engrafted and generated more subcutaneous tumors than the parental adherent melanoma cells. Specifically, when cells (5 × 10^5^ cells/200 μl/mouse) were transplanted, tumor developed in 4/6 (shperoid-derived cells) vs 2/4 (adherent cells) injected mice (Fig. 2c, left panel) and we noticed that spheroid-derived cells resulted in a slightly faster appearance of the tumors. As shown in Fig. 2c, left panel, tumor take rate was 1/6 vs 0/4 at 2 weeks; 3/6 vs 1/4 at 3 weeks; 4/6 vs 2/4 at 4 weeks in spheroid-derived cells and in adherent cells, respectively. Once engrafted, all the generated tumors grew rapidly, the growth rate (proliferation rate) of melanosphere-derived tumors was similar to that of adherent cell-derived tumors (Fig. 2c, right panel).

On the other hand, 5 × 10^4^ spheroid-derived cells developed a tumor only in 1/6 injected mice while adherent cells were not tumorigenic (0/6).

### Flow cytometry analysis of CD271 staining on A375 adherent cells vs spheroid-derived cells

**Figure 3:**
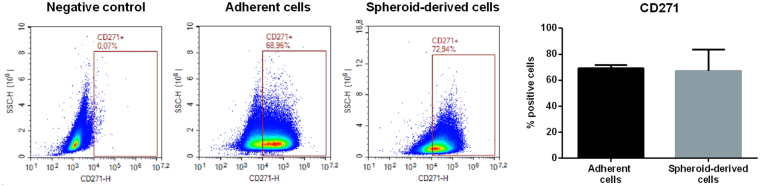
Flow cytometry analysis of CD271 staining on A375 adherent cells vs spheroid-derived cells. The proportion of CD271-positive cells was measured in A375 adherent cells or their spheroid-forming counterpart. Gating strategy was based on negative controls, without incubation with the specific antibody. Data were analyzed by unpaired t-test, and are represented by mean values ± SEM. One representative of four different experiments is shown.

The reliability of surface markers expression in different types of tumors, including melanoma, has recently become a matter of debate^15,16^. Based on this observation, experiments were then performed to verify whether the surface marker CD271 could be considered a reliable stem cell marker in A375 cells. To this purpose, a cytofluorimetric analysis was performed in order to assess the percentage of CD271-positive cells in both adherent and spheroid culture conditions. Surprisingly, the data obtained clearly show that A375 melanospheres are not enriched in CD271-positive cells, since the percentage of expression of this marker was similar (about 70%) in spheroids and in adherent cells (Fig. 3). These data are in agreement with those by Boyle and coworkers^15^ showing that CD271 expression in patient-derived melanoma cells is unstable and unlinked to tumorigenicity.

Taken together, these results confirm that, in our experimental conditions, A375 spheroids are highly enriched in CSCs that express pluripotent embryonic stem cell markers, are highly invasive and and have a higher tumor forming capability *in vivo* (when inoculated in nude mice) with respect to parental adherent cells.

### Intracellular autofluorescence, associated with ABCG2 expression, is a novel marker for A375 CSCs

**Figure 4:**
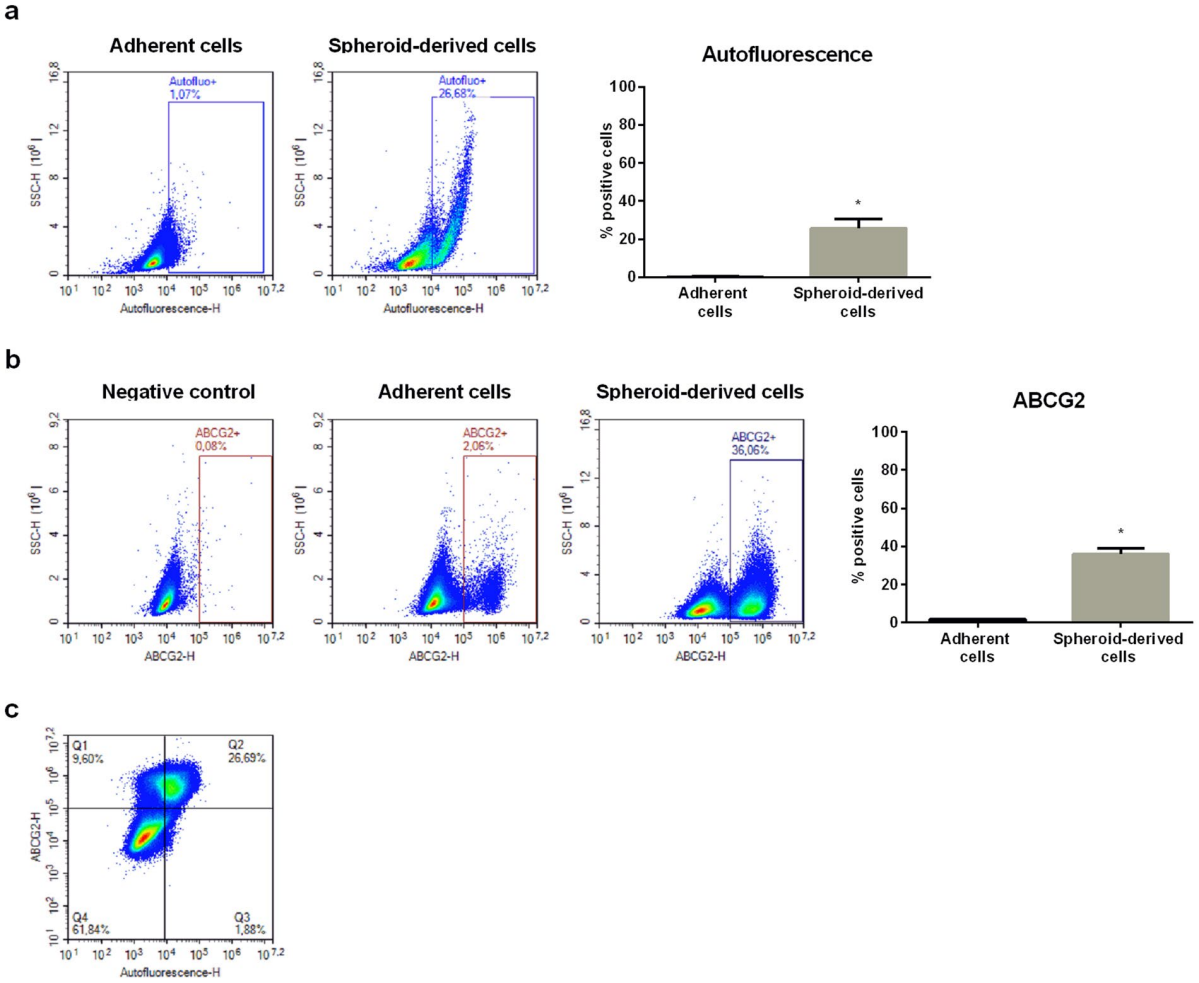
Intracellular autofluorescence, associated with ABCG2 expression, is a novel marker for A375 CSCs. (**a**) Cellular autofluorescence (λ_em_ 532 nm) was measured in A375 adherent and spheroid-derived cells by flow cytometry, observing the presence of two cell populations. Data were analyzed by unpaired t-test, and are represented as mean values ± SEM (*p < 0.05 vs Adherent cells). One representative of four different experiments is shown. (**b**) The proportion of ABCG2-positive cells was measured in adherent or melanosphere-derived cells. Gating strategy was based on negative controls, without incubation with the specific antibody. Data were analyzed by unpaired t-test and are represented by mean values ± SEM (*p < 0.05 vs Adherent cells). One representative of four different experiments is shown. (**c**) The relationship between autofluorescence and ABCG2 expression in melanosphere-derived cells was analyzed by flow cytometry. Gating strategy was based on negative controls, without incubation with the specific antibody (left panel). Moreover, highly autofluorescent cells were gated and analyzed for their ABCG2 positivity (right panel). One representative of three different experiments is shown.

It is now accepted that surface CSCs markers do not represent a sensitive method to isolate and characterize these cells^15,16^. The results reported above, showing that A375 melanospheres are not enriched in the CD271 marker, are in line with these observations. Miranda-Lorenzo and coworkers, in epithelial tumors, identified cells with autofluorescent properties showing CSCs features and phenotypes. Autofluorescence was reported to be due to the presence of riboflavin (vitamin B2) in membrane-bounded cytoplasmic vesicles coated with the ATP-dependent transporter ABCG2. The authors concluded that autofluorescence can be considered a reliable marker to be used for the isolation of CSCs from the bulk of a tumor mass^17^. Based on this observation, by cytofluorimetric analysis we analyzed the autofluorescent properties (excitation with a standard blue laser at 488 nm and emission at 532 nm) of A375 cells, both adherent and spheroid-derived cells, and their relationship with the expression of ABCG2. We observed that a subpopulation of autofluorescent cells (about 26%) is present in the A375 spheroid cultures but not in adherent cells (Fig. 4a). Moreover, we found that a similar percentage of cells expresses the ABCG2 transporter (Fig. 4b), previously shown to represent a reliable marker of stemness in metastatic lesions from melanoma patients^36^. Interestingly, by analyzing the possible relationship between cell autofluorescence and the transporter expression, we could demonstrate that cells with higher autofluorescence are also positive for the expression of ABCG2 (about 26%) (Fig. 4c). Thus, we conclude that the evaluation of the intrinsic autofluorescent properties together with the expression of the ABCG2 stem cell marker, can be considered a reliable method for the detection and isolation of CSCs. Our data demonstrate that a subpopulation of cells endowed with these features is present also in A375 melanoma cells.

### δ-TT and vemurafenib differentially affect the formation of primary A375 melanospheres

Based on their role in drug resistance and tumor relapse, CSCs are considered a very attractive molecular target for novel anticancer compounds, both synthetic and natural. It is widely accepted that conventional therapies preferentially kill differentiated or differentiating cells, which form the bulk of the tumor but fail to affect the stem cell subpopulation of cancers^37^. We previously reported that vitamin E-derived δ-TT significantly induces apoptosis in melanoma cells, through activation of the ER stress prodeath pathway, both *in vitro* and *in vivo*^31^. Experiments were then performed to investigate the ability of δ-TT to affect the A375 stem cell subpopulation and to compare it with that of vemurafenib, the classical targeted therapeutic compound utilized for the treatment of BRAF mutated melanomas.

**Figure 5:**
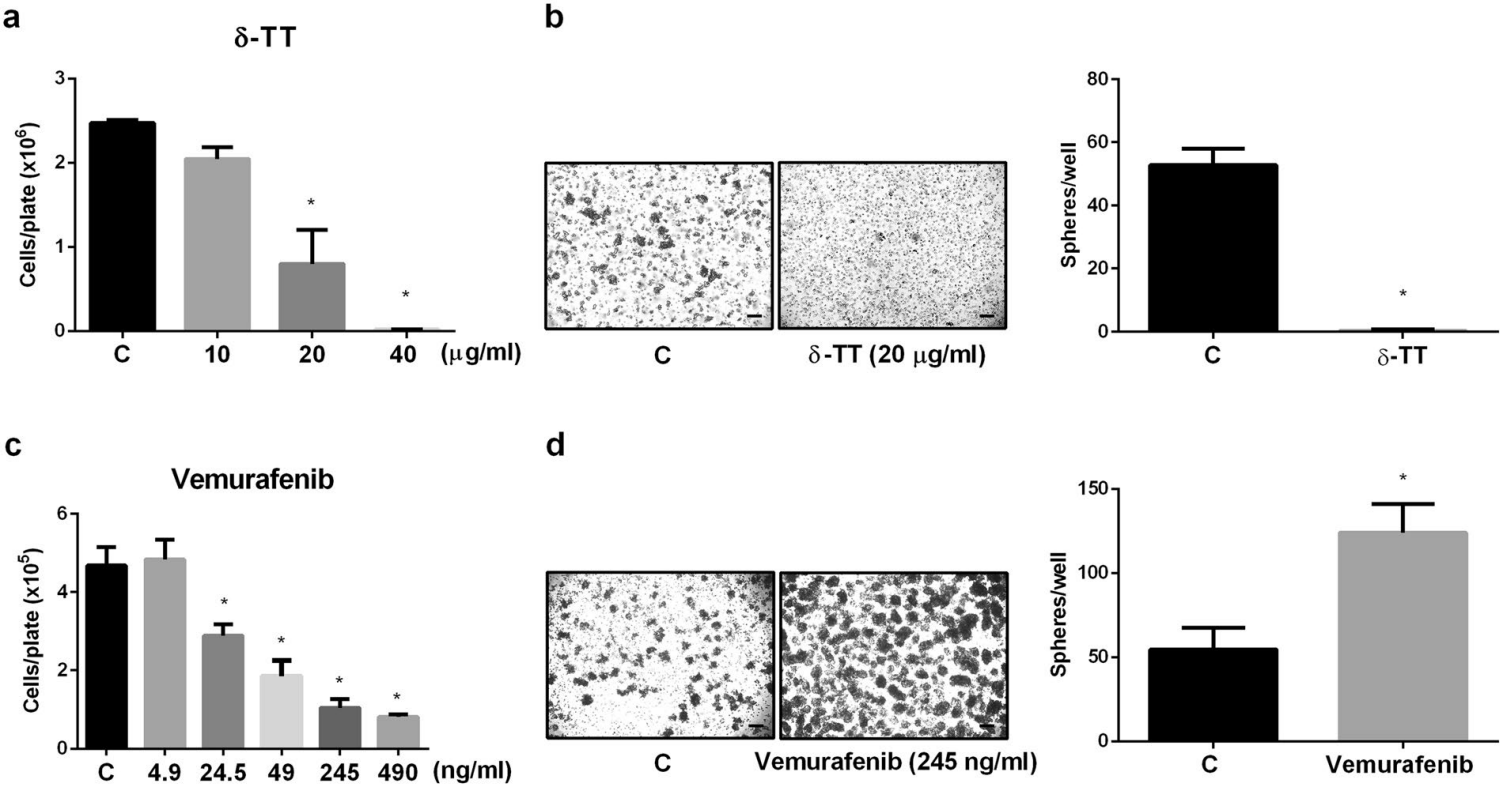
δ-TT and vemurafenib differentially affect the formation of primary A375 melanospheres. (**a**) Adherent cells were treated with scaling doses (10–40 μg/ml) of δ-TT for 18 h, then cell proliferation was assessed by cell counting. Data represent mean values ± SEM and were analyzed by Dunnett’s test after one-way analysis of variance (*p < 0.05 vs C). One representative of three different experiment is shown. (**b**) In order to assess the ability of δ-TT to affect the formation of melanospheres, adherent cells were treated with the compound (20 μg/ml) for 18 h, replated in stem cell medium and left to grow for up to 15 days. Melanospheres were photographed by a 4 × /1.4 objective lens (left panel, scale bar, 75 μm), and counted for each well (right panel). Data were analyzed by unpaired t-test, and are represented as mean values ± SEM (*p < 0.05 vs C). One representative of three different experiments is shown. (**c**) Vemurafenib treatment was performed in order to compare the effect of δ-TT with the standard therapy. Adherent cells were treated with scaling doses of this compound (4.9–490 ng/ml; 10–1000 nM) for 96 h and then cell proliferation was assessed by counting the cells. Data represent mean values ± SEM and were analyzed by Bonferroni’s test after one-way analysis of variance (*p < 0.05 vs C). One representative of three different experiments is shown. (**d**) Adherent cells were treated with 245 ng/ml (500 nM) of this compound for 96 h, then cells were replated in stem cell medium, and left to grow for up to 15 days. Melanospheres were photographed by a 4 × /1.4 objective lens (left panel, scale bar, 75 μm), and counted for each well (right panel). Data were analyzed by unpaired t-test, and are represented as mean values ± SEM (*p < 0.05 vs C). One representative of three different experiments is shown. C, controls.

A375 adherent cells were treated either with δ-TT (10–40 μg/ml) for 18 h or with vemurafenib (4.9, 24.5, 49, 245, 490 ng/ml; 10–1000 nM) for 96 h in standard culture conditions; cells escaping the treatment with δ-TT (20 μg/ml) or with vemurafenib (245 ng/ml; 500 nM) were then replated in stem cell medium, up to 15 days, without treatment. We observed that both δ-TT (Fig. 5a) and vemurafenib (Fig. 5c) significantly and dose-dependently decrease cell proliferation. Moreover, cells escaping the antitumor activity of δ-TT (20 μg/ml) were completely devoid of the ability to form melanospheres (Fig. 5b); on the other hand, cells that have escaped vemurafenib treatment (245 ng/ml) showed a significantly higher ability to form melanospheres with respect to control cells (Fig. 5d). These data indicate that, in contrast with standard treatments (e.g., vemurafenib), δ-TT exerts its antitumor effects on melanoma cells also by targeting the CSCs subpopulation.

### δ-TT impairs the growth of A375 melanospheres

**Figure 6:**
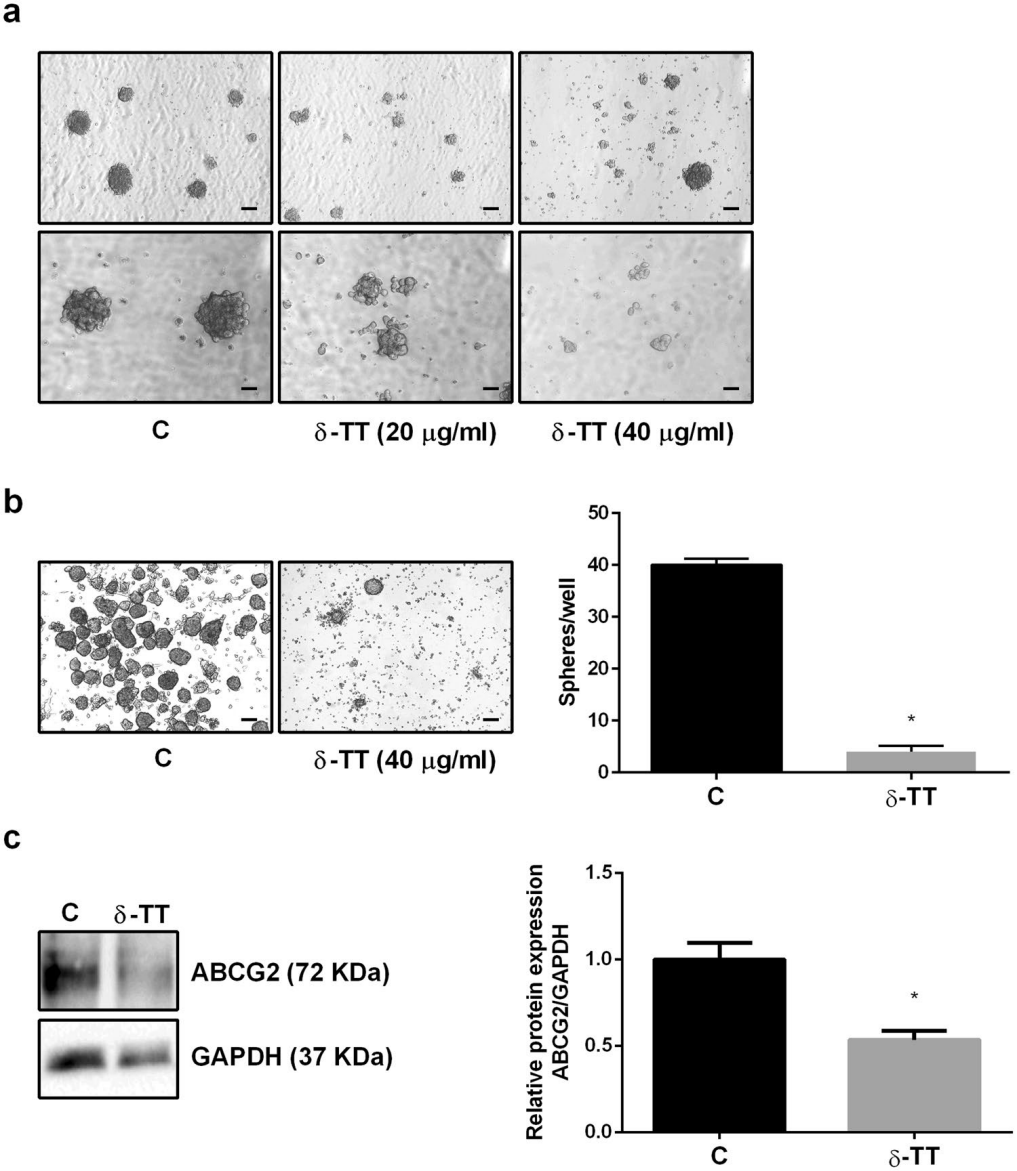
δ-TT impairs the growth of A375 melanospheres. (**a**) A375 melanospheres were collected and treated with 20 or 40 μg/ml of δ-TT for 5 days. Spheres were then photographed with a 4 × /1.4 (upper panels, scale bar, 75 μm) or a 10 × /1.4 (lower panels, scale bar, 30 μm) objective lens, in order to capture number, size and morphology changes. (**b**) In order to assess the effects of δ-TT on melanosphere growth, melanosphere-derived cells were plated (3 × 10^4^ cells/well), and treated (40 μg/ml) during melanosphere formation, once/week for two weeks. At the end of the treatment, spheres were photographed with a 4 × /1.4 objective lens (scale bar, 75 μm), and counted for each well. Data were analyzed by unpaired t-test, and are represented by mean values ± SEM (*p < 0.05 vs C). (**c**) Protein extracts from the same experiment (**b**) were collected, and the expression of ABCG2 was analyzed by Western blot. Evaluation of GAPDH expression was used as a loading control. Densitometric analysis was performed with ImageJ software, data were analyzed by unpaired t-test and are represented by mean values ± SEM (*p < 0.05 vs C). One representative of three different experiments is shown. C, controls. Cropped gels/blots are displayed. Original uncropped Western blots were reported in the Supplementary Figure S1B.

Experiments were performed to confirm a direct anticancer effect of δ-TT on melanoma CSCs. A375 pre-formed melanospheres were plated and after 48 h were treated with δ-TT (20 or 40 μg/ml) for 5 days. Fig. 6a shows that δ-TT does not induce the complete disaggregation of pre-formed melanospheres; on the other hand, δ-TT-treated melanospheres are characterized by reduced dimensions, border irregularity and lack of compactness in contrast with control spheres. This might be related to a reduced accessibility of δ-TT to CSCs in melanospheres than in adherent cells (as observed in Fig. 5b). Likely, δ-TT acts on the outer cells of the spheroids, leading to a change of their morphology, before reaching the inner cells of the spheres. Thus, we hypothesized that, in these experimental conditions, higher doses or longer time intervals of treatment would be required to observe a significant effect of the compound on pre-formed melanospheres.

Based on these results, A375 melanospheres were disaggregated and the obtained single cells (supposed to have the same sphere-formation potential) were plated in stem cell medium; during sphere formation, 48 hours after plating, they were treated with δ-TT (40 μg/ml) for 2 weeks, twice/week. At the end of the treatment, the number of spheres that could form in each well was significantly reduced in δ-TT-treated cells, confirming our previous hypothesis about the reduced accessibility of δ-TT on pre-formed melanospheres. Moreover, the dimensions of treated melanospheres were more variable compared to those of non-treated spheres (Fig. 6b). In line with these results, we could further demonstrate that the expression level of the ABCG2 marker was significantly lower in δ-TT-treated melanospheres than in control spheres (Fig. 6c).

Taken together, these results indicate that CSCs can represent an effective target for the antitumor activity of δ-TT in melanoma.

## Discussion

The major problem regarding melanoma standard treatments (e.g., chemotherapy, targeted therapies) is still represented by the poor clinical outcome due to development of resistance to drugs^3,38–40^. More recently, immune checkpoint inhibitors were developed with the aim to increase the anti-tumor immune response. By counteracting the negative activity of melanoma cells on the immune system, monoclonal antibodies anti cytotoxic-T-lymphocyte-associated antigen 4 (CTLA-4), such as ipilimumab, and anti programmed cell death-1 (PD-1), such as nivolumab and pembrolizumab, were introduced in the clinics for the treatment of advanced melanomas^41,42^. However, despite an initial enthusiasm due to the general improvement in patient outcome, development of primary and secondary resistance to these therapies has been recently reported^43–46^. Thus, the discovery of novel therapeutic strategies (e.g., novel drugs and/or combination regimens) is urgently needed for patients with advanced melanoma.

Based on their key role in the mechanisms of drug resistance, CSCs are now considered a promising effective target for novel therapeutic strategies for cancer^11,12^. So far, several compounds, both synthetic and natural, have been reported to exert a significant antitumor activity by targeting cancer ‘stemness’, besides decreasing the viability of the bulk population of cells, in different types of tumors^25,47^.

We recently reported that vitamin E-derived δ-TT exerts a significant proapoptotic activity in melanoma cells (while sparing normal melanocytes), through the activation of the endoplasmic reticulum stress signaling pathway^31^. Based on these observations, aim of this work was to: 1) identify and characterize the CSC subpopulation in melanoma cell lines, and 2) investigate whether δ-TT might exert its antitumor activity against melanoma cells by targeting this aggressive subpopulation of tumor cells.

We found that both A375 and BLM melanoma cells express the surface stem cell markers ABCB5 and CD44, as evaluated by Western blotting and by immunofluorescence analysis; on the other hand the stem cell marker CD271 is expressed only in A375 cells. More importantly, A375 cells are able to form melanosphere when cultured in appropriate conditions, while this peculiar feature of stemness is absent in BLM cells. Based on these observations, we concluded that a subpopulation of CSCs may not present in the BLM cell line (although further studies are required to definitely confirm this hypothesis), and focused our further experiments on A375 cells. We demonstrated that these cells express the embryonic stem cell markers SOX2 and KLF4 by real-time PCR and possess a significant invasive behavior when compared to adherent cells. Importantly, A375-derived spheroid cells were more efficient in terms of engraftment and tumor generation than the parental adherent cells. Finally, to confirm the relevance, in melanoma cells, of the expression of the surface marker CD271, a cytofluorimetric analysis was performed in order to assess the percentage of CD271-positive cells in both adherent and spheroid derived cells. Surprisingly, we could observe that A375 melanospheres are not enriched in CD271-positive cells, since the percentage of expression of this marker was similar (about 70%) in spheroids and in adherent cells. Taken together, these data are in line with previous observations supporting a peculiar heterogeneity of CSCs in different melanoma tumors^16^ and indicating that CD271 expression on melanoma cell surface does not represent a reliable marker of cell stemness^15^. On the other hand, according to other authors, CD271 plays a crucial role in maintaining tumorigenicity and stem-like features of melanoma cell lines, including A375 cells^48^. This issue needs to be further exploited.

In general, the data here reported agree with the concept that the expression of surface markers is not exclusively linked to a specific functional cancer stem cell phenotype; moreover, their levels of expression change according to the experimental model in which they are investigated (tissues *vs*. cell cultures). As outlined above, with the aim to develop novel methods for the identification and isolation of the cancer stem cell subpopulation, Miranda-Lorenzo and coworkers^17^ identified cells with autofluorescent properties showing CSCs features and phenotypes in different types of epithelial cancer cells. Autofluorescence was reported by these authors to be due to the presence of riboflavin (vitamin B2) in membrane-bounded vesicles that are coated with the ATP-dependent transporter ABCG2. In this paper, by cytofluorimetric analysis, we could observe that a subpopulation of cells expressing high autofluorescence is present in the A375 spheroid cultures but not in adherent cells, and that a similar percentage of spheroid derived cells express the ABCG2 transporter. Interestingly, we could also demonstrate that cells with higher autofluorescence are also positive for the expression of ABCG2. Thus, in line with Miranda-Lorenzo and coworkers, the results here reported support that the evaluation of intrinsic autofluorescent properties could be considered a reliable method for the detection and isolation of CSCs in melanoma cells. Interestingly, ABCG2 has been reported to be expressed in the majority of metastatic melanoma tissue specimens and its levels of expression correlated with patient survival^36^.

Taken together, our data demonstrate that a subpopulation of cells expressing stem-like traits is present in A375 melanoma cells. These cells display the mostly peculiar stemness features: ability to form tumor spheres when cultured in appropriate conditions, expression of pluripotent embryonic stem cell markers, highly invasive behavior, tumor formation capacity when inoculated in nude mice, and an intrinsic autofluorescent phenotype.

The presence of CSCs in different melanoma cell lines and tumors was previously reported^16,34,49^ and was suggested to serve as an effective therapeutic target for melanoma treatment^50^. However, to the authors’ knowledge, this is the first report in the literature demonstrating that also melanoma CSCs, in addition to epithelial cancer cells as previously described^17^, can be identified and characterized on the basis of their intrinsic autofluorescence.

Based on these results, we then sought to investigate whether δ-TT might affect the CSCs subpopulation. It is well known that standard therapies, such as chemotherapy or targeted therapies, exert their anticancer activity by suppressing the viability of the bulk of tumor mass, while sparing CSCs, in most of tumors, including melanoma^37,51,52^. A375 cells were treated either with vemurafenib, the standard treatment for BRAF-mutant melanomas, or with δ-TT; at the end of the treatments, treatment-escaping cells were replated in stem cell medium. Interestingly, we could observe that cells surviving and escaping vemurafenib treatment have a significantly higher ability to form melanosperes than control cells; on the other hand, cells escaping from δ-TT are completely devoid of the ability to form melanospheres. These data confirm the notion that targeted therapies preferentially affect the proliferation of the bulk of tumor cells, while sparing CSCs; more importantly, these results clearly demonstrate that the vitamin E derivative δ-TT is able to eradicate the CSC subpopulation in melanoma cells. We could further confirm this observation by demonstrating that treatment of well-formed melanospheres with δ-TT, even if it does not seem to induce their disaggregation, is able to reduce their dimensions and to induce irregularity of the borders and loss of compactness. This is possibly related to the reduced accessibility of the compound to CSCs in the inner part of the melanospheres. Moreover, δ-TT treatment of single cells derived from disaggregated spheres and cultured in stem cell medium significantly hampered the ability of these cells to form melanospheres; interestingly, the expression levels of the ABCG2 marker was significantly reduced in δ-TT-treated with respect to control melanospheres. We previously showed that δ-TT significantly decreases the viability of melanoma cells (while sparing normal melanocytes) by triggering the endoplasmic reticulum stress pathway^31^. The data here reported demonstrate that, besides its proapoptotic activity on the bulk of melanoma cells, δ-TT also targets the CSC subpopulation of these tumor cells. To this purpose, it must be underlined that the bioavailability and safety of oral administration of δ-TT in healthy subjects have been recently reported^53,54^. In line with our data, δ-TT was recently shown to inhibit pancreatic cancer stem-like cells thus preventing cancer metastasis^55,56^. Another vitamin E tocotrienol isomer, γ-TT, was reported to specifically target the CSC subpopulation in different cancer cells (colorectal, breast, prostate)^57–60^. This ability to counteract tumor growth by targeting both the bulk tumor cells and the CSC subpopulation has previously been reported for other natural compounds^21–25^.

The molecular mechanisms underlying the inhibitory activity of δ-TT on cancer stem cells are still poorly understood. The cyclooxygenase-2/prostaglandin E2 (COX-2/PGE2) axis was reported to be deeply involved in the growth of the CSC subpopulation in several cancer cell lines^61^, through activation of the Wnt/β-catenin signaling pathway^62^. Interestingly, Zhou and coworkers^63^ recently demonstrated that COX-2 is overexpressed in highly aggressive, invasive and metastatic melanoma cells. More importantly, δ-TT and its metabolite δ-TT-13′-COOH were shown to suppress COX-2 expression in colon cancer cells^64^. These data strongly support the hypothesis that δ-TT might exert its antitumor activity against CSCs also in melanoma cell lines, through inhibition of COX-2 expression/activity.

In addition, γ-TT was reported to eliminate enriched CSCs in drug resistant breast cancer cell lines, by suppressing the expression of Stat-3 (signal transducer and activator of transcription 3) signaling mediators^65^. A better understanding of the molecular mechanisms underlying the ability of δ-TT to suppress CSCs in tumors will further support its crucial role as an antitumor compound able to eradicate this very aggressive tumor cell subpopulation, involved in drug resistance development and tumor relapse.

In summary, the data here reported demonstrate that a subpopulation of stem cells is present in the A375 melanoma cell line. These cells are able to form melanospheres when cultured in appropriate conditions, express the embryonic SOX2 and KLF4 stem cell markers, are associated with a high invasive capacity, are more efficient than adherent cells in terms of engraftment and tumor formation when inoculated into nude mice, and are characterized by intracellular autofluorescence associated with ABCG2 expression. Moreover, the vitamin E derivative δ-TT (but not vemurafenib) specifically targets melanoma CSCs, as demonstrated by its ability to significantly impair both the formation and the growth of melanospheres. These results could provide new insights into the potential clinical utility of this compound with the aim to overcome chemoresistance and, therefore, to improve the therapeutic options for a highly aggressive tumor, such as melanoma.

## Methods

### Cell cultures

The A375 human melanoma cell line was purchased from American Type Culture Collection (ATCC, Manassas, VA, USA). The BLM human melanoma cell line was kindly provided by Dr. G. N. van Muijen (Department of Pathology, Radbound University Nijmegen Medical Center, Nijmegen, The Netherlands). This cell line is a subline of BRO melanoma cells isolated from lung metastases after subcutaneous inoculation of nude mice with BRO cells^66^. Original stocks of cells were stored frozen in liquid nitrogen; after resuscitation, cells were kept in culture for no more than 10–12 weeks.

A375 and BLM cell lines were routinely grown in DMEM medium supplemented with 7.5% and 10% FBS, respectively, glutamine (1 mmol/l) and antibiotics (100 IU/ml penicillin G sodium and 100 μg/ml streptomycin sulfate) and cultured in humidified atmosphere of 5% CO_2_/95% air. Cells were detached through trypsin-EDTA solution and passaged once/week.

For melanosphere formation assays, cells were seeded at 5 × 10^5^ cells per 25-cm^2^ flask and cultured in Euromed-N serum-free medium (Euroclone, Pero, Mi, Italy), supplemented with EGF (10 ng/ml), FGF-2 (10 ng/ml), 1% N2 supplement and antibiotics. Floating tumor spheres formed within 5–7 days. To enrich melanoma cell culture of initiating-tumor cells, floating spheres were harvested and passaged every 15 days: the supernatant in the flask was recovered and centrifuged for 5 min at low speed (100 × g), to allow the separation of voluminous melanospheres from single cell-suspension. Spheres were then resuspended in fresh Euromed-N mixed 50% with melanospheres conditioned medium, mechanically dissociated by pipetting, and plated in new 25-cm^2^ flasks. Euromed-N was added every 72 h to the culture.

### Materials

For Western blot and immunofluorescence analyses, the following primary antibodies were utilized: ABCB5 (5H3C6, LifeSpan BioSciences, Inc., Seattle, WA); CD44 (156-3C11) and CD271 (D4B3, Cell Signaling Technology Inc., Boston, MA); ABCG2 (B1, Santa Cruz Biotechnology, Inc., Dallas, TX). HRP-conjugated secondary antibodies were from Cell Signaling Technology Inc. Alexa Fluor 488 and 594-conjugated secondary antibodies were from Molecular Probes Inc. (Eugene, OR).

For flow cytometry analyses, PE-CD271 conjugated antibody (ME20.4) was from eBioscience (San Diego, CA), and Alexa Fluor 405-ABCG2 conjugated antibody (MM0047-2J39) was from Novus Biologicals (Littleton, CO).

The BRaf^V600E^ inhibitor Vemurafenib was from Selleckchem (Houston, TX).

δ-TT was purified from a commercial extract of Annatto (*Bixa orellana*) seeds (kindly provided by DeltaGold, American River Nutrition Inc., Hadley, MA), as previously described^31,67^, and aliquots of 50 mg in anhydrous dimethylsulfoxide (Sigma-Aldrich, Milano, Italy) stored at − 20 °C until testing (50 mg/ml).

### Western blot assays

To investigate the expression of stem cells markers, adherent A375 and BLM cells were processed as described^31^. Protein extracts (15–20 μg) were resuspended in reducing sample buffer (Bio-Rad Laboratories, Segrate, Mi, Italy) and heated at 95% for 5 min. Following electrophoretic separation by SDS-PAGE, proteins were transferred onto nitrocellulose membranes. After blocking, membranes were incubated with the primary antibodies against ABCB5 (1:1000), CD271 (1:1000) and CD44 (1:1000). Detection was done using HRP-conjugated secondary antibodies and enhanced chemiluminescence ECL-Prime reagents.

To assess the expression of ABCG2 following δ-TT treatment, adherent A375 cells were seeded (5 × 10^5^ cells/dish) in 10-cm dishes and treated with δ-TT (20 μg/ml) or vehicle for 18 h, then protein extraction and Western blot assay were performed as described above. After blocking, membranes were incubated with the primary antibody against ABCG2 (1:1000). Detection was done using HRP-conjugated secondary antibodies and enhanced chemiluminescence ECL-Prime reagents.

To analyze the expression of ABCG2 in the spheroid cultures after δ-TT treatment, melanospheres were mechanically disaggregated by pipetting to reach a single cell suspension. Cells were then seeded (3 × 10^4^ cells/well) in a 12-multiwell plate. After 48 h, cells were treated with δ-TT (40 μg/ml) or vehicle once a week for two weeks. The supernatant was then collected and centrifuged (100 × g), and protein preparations were processed for Western blotting, as described above.

GAPDH expression was analyzed as a loading control; the primary GAPDH antibody concentration was 1:2000.

### Immunofluorescence assays

A375 and BLM cells were seeded at 3 × 10^4^ cells/well on polylysine-coated 13-mm diameter coverslips, and left to grow for 48 hours. Then, cells were fixed with 3% paraformaldehyde in 2% sucrose-PBS and stained with ABCB5, CD44 or CD271 antibodies, followed by secondary antibodies and DAPI. Labeled cells were then examined and photographed with MetaVue software, under a Zeiss Axiovert 200 microscope linked to a Coolsnap Es CCD camera (Roper Scientific-Crisel Instruments, Roma, Italy), with a 63 × /1.4 objective lens.

### RT-real time PCR

Quantative PCR was performed to verify the expression of embryonic stem cell marker mRNAs (SOX2 and KLF4). A375 cells, in both adherent and spheroid conditions, were harvested and total RNA was extracted using the Direct-zol RNA miniprep extraction kit (Zymo research, Irvine, CA), following the manufacturer’s protocol. RNA concentrations were assessed using Nanodrop 2000 (Thermo Fisher Scientific, Waltham, MA).

Specific sets of primers for SOX2 and KLF4 cDNAs were designed and synthesized (Sigma-Aldrich, Milano, Italy), according to Miranda-Lorenzo and coworkers^17^. Real-time PCR was performed as previously described^68^. 18 S levels were used as housekeeping controls. The amplification of gene expression was quantified using the comparative threshold-cycle (DDCt) method.

### Invasion assays

A375 adherent and spheroid-derived cells were assayed for their invasive ability through a matrigel invasion assay. A 24-multiwell plate was coated with Matrigel (200 μl, BD Biosciences, Milano, Italy) and 100 cells were seeded for each well. Adherent cells, and spheroid-derived cells obtained by mechanical disaggregation (pipetting) of melanospheres, were plated in DMEM supplemented with 7,5% FBS, and left to grow for two weeks. At this time point, cells were photographed under a Zeiss Axiovert 200 microscope linked to a Coolsnap Es CCS camera, with a 4 × /1.4 objective lens.

### *In vivo* assessment of melanosphere-derived cell tumorigenicity

A375 adherent and spheroid-derived cells were assayed for their ability to form tumors in nude mice. Six weeks-old athymic nude mice were obtained from Envigo (Udine, Italy) and housed under specific pathogen-free conditions in isolated vented cages. Procedures involving animals and their care were conducted at the Istituto di Ricerche Farmacologiche Mario Negri in conformity with institutional guidelines in compliance with national (Legislative Decree 26/2014) and international (EEC Council Directive 2010/63) laws and policies, in line with the guidelines for the welfare and use of animals in cancer research^69^. The studies were approved by the Mario Negri Institute Animal Care and Use Committee. Melanosphere-derived cells, obtained by disaggregation through pipetting, and adherent cells were diluted in HBSS and subcutaneously injected in mice (5 × 10^4^ or 5 × 10^5^ cells/200 μl, 4–6 animals/group). Tumor take (initiation) was assessed by palpation (twice a week), and tumor growth was monitored weekly by measuring tumor size with a Venier caliper and calculating tumor volume (mm^3^) as [length (mm) x width^2^ (mm^2^)/2]^70^. When the successful engrafted tumors reached a size of approximately 500 mm^3^, mice were sacrificed by carbon dioxide inhalation followed by cervical dislocation.

### Flow cytometry analyses

Flow cytometry analyses were performed in A375 adherent cells and in melanosphere-derived cells, in order to assess the expression of CD271 and ABCG2, and the autofluorescence level of the cells. Melanospheres were disaggregated by accutase (Euroclone) incubation. Single cells were resuspended in ice cold PBS containing 2% FBS (5 × 10^5^ cells and 2.5 × 10^5^ cells, respectively), and incubated with PE-conjugated anti-CD271 antibody or/and Alexa Fluor 405-conjugated anti-ABCG2 antibody. Cells were then washed with ice cold PBS and analyzed with excitation wavelengths of 488 (CD271) and 405 (ABCG2) nm, and a collection filter of 585/40 and 445/45 nm, respectively.

The autofluorescence analysis was performed without labeling the cells, using an excitation wavelength of 488 nm and the FITC collection filter (530/30), as previously described^17^. The flow cytometry analysis was performed with a Novocyte3000 instrument (ACEA Biosciences, San Diego, CA). Data were analyzed with Novoexpress software.

### Proliferation assays

In order to assess the effect of δ-TT and vemurafenib on cell proliferation, A375 cells were seeded in 6-cm dishes (1,5 × 10^5^ and 3 × 10^4^ cells/dish, respectively). Cells were then treated with vemurafenib (4.9, 24.5, 49, 245, 490 ng/ml; 10–1000 nM) for 96 h, δ-TT (10, 20, 40 μg/ml) for 18 h, or vehicle. At the end of the treatments, cells were harvested by trypsinization and counted by hemocytometer.

### Melanosphere formation assays

The spheroidogenic potential of melanoma cells was monitored after treatment with vemurafenib, δ-TT or vehicle. Adherent A375 cells were seeded in 10-cm dishes (8 × 10^4^ and 10^6^ cell/dish) and treated with 245 ng/ml (500 nM) vemurafenib (96 h) or 20 μg/ml δ-TT (18 h). Treatment-escaping cells were collected and seeded at 3 × 10^4^ cells/well in stem cells medium, in a 12-multiwell plate. Fresh medium was added every 72 h, and melanospheres were grown for up to two weeks: at this time point, melanospheres were photographed with a 4 × /1.4 objective lens, and counted for each well.

### Melanosphere treatments

To examine the effect of δ-TT on melanosphere morphology, A375 melanospheres were collected and plated in a 12-multiwell plate. After 48 h they were treated with δ-TT (20 or 40 μg/ml) or vehicle for 5 days. At the end of the treatment, spheres were photographed with a 4 × or 10 × /1.4 objective lens.

In order to assess the effect of δ-TT on melanosphere number, spheres were disaggregated by pipetting, and single cells were counted and seeded at 3 × 10^4^ cells/well in a 12-multiwell plate. After 48 h, cells were treated with δ-TT (40 μg/ml) or vehicle once a week for two weeks. Melanospheres for each well were then counted and photographed with a 4 × /1.4 objective lens.

### Statistical analysis

Statistical analysis was performed with a statistic package (GraphPad Prism5, GraphPad Software San Diego, CA, USA). Data are represented as the mean ± SEM of three-four independent experiments. Differences between groups were assessed by t-test or by one-way analysis of variance (ANOVA) followed by Dunnet’s or Bonferroni’s test. A p value < 0.05 was considered statistically significant.

### Data availability

The datasets generated during and/or analyzed during the current study are available from the corresponding author on reasonable request.

## Electronic supplementary material

Supplementary information
