# Supplementary material for: Targeting melanoma stem cells with the Vitamin E derivative δ-tocotrienol
Source: Sci Rep. 2018 Jan 12;8:587. doi: 10.1038/s41598-017-19057-4 (PMC5766483; doi:10.1038/s41598-017-19057-4)
Supplement: Supplementary file 1 — Supplementary information [file 41598_2017_19057_MOESM1_ESM.pdf]

Supplementary information

Targeting autofluorescent melanoma stem cells with the Vitamin E derivative δ-tocotrienol

Monica Marzagalli[1†], Roberta Manuela Moretti[1†], Elio Messi[1], Marina Montagnani Marelli[1], Fabrizio Fontana[1], Alessia Anastasia[2], Maria Rosa Bani[2], Giangiacomo Beretta[3] & Patrizia Limonta[1,*]

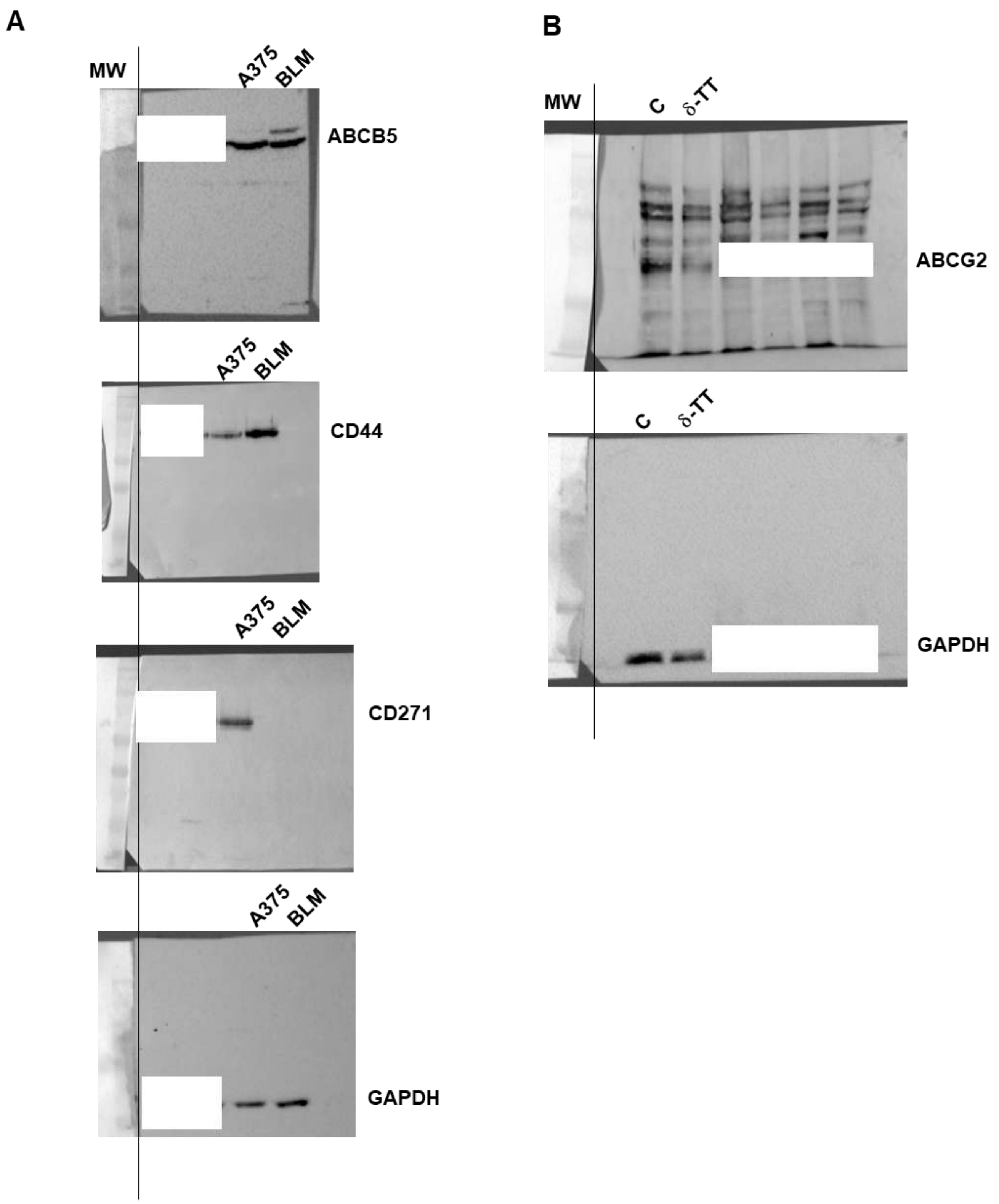

Figure S1. Original uncropped Western blots of the images reported in Fig. 1b (A) and in Fig. 6c (B). The white shadows covered samples out of this study.
